# *QuickStats:* Percentage of Adults Aged ≥20 Years Told Their Cholesterol Was High Who Were Taking Lipid-Lowering Medications,* by Sex and Age Group — National Health and Nutrition Examination Survey, 2005–2006 to 2015–2016

**DOI:** 10.15585/mmwr.mm6727a6

**Published:** 2018-07-13

**Figure:**
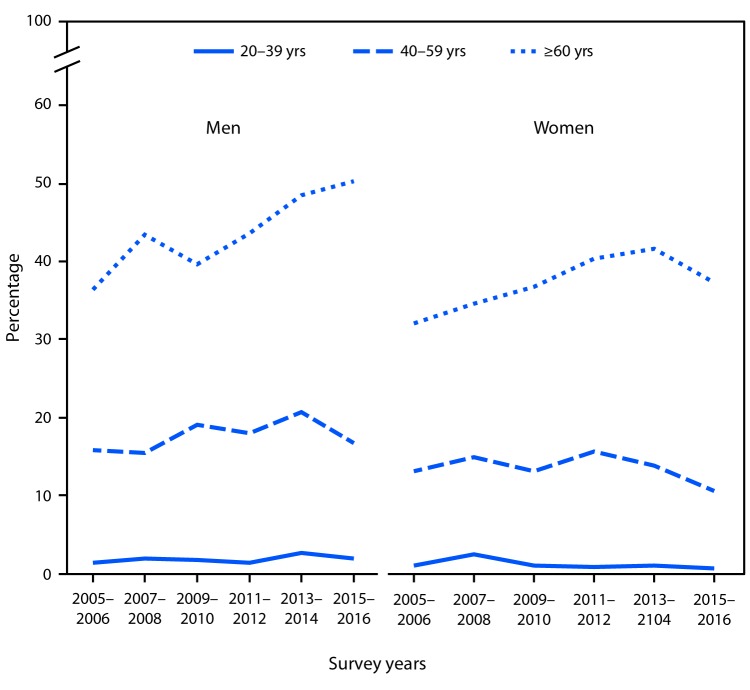
The percentage of men told by a health professional that their cholesterol was high who were taking lipid-lowering medications increased from 36% in 2005–2006 to 50% in 2015–2016 among those aged ≥60 years but not among those aged 20–39 years (1% to 2%) or 40–59 years (16% to 17%). The percentage taking lipid-lowering medications also increased (from 33% to 38%) among women aged ≥60 years but not among women aged 20–39 years (1% to 0.7%) or 40–59 years (from 13% to 11%). For each survey year from 2005–2006 to 2015–2016, the percentage of both men and women with high cholesterol taking lipid-lowering medications was higher among those aged ≥60 years than those in younger age groups.

**Sources: **Carroll MD, Mussilino ME, Wolz M, Srinivas PR. Trends in apolipoprotein B, non–high-density lipoprotein, and low-density lipoprotein for adults 60 years and older by use of lipid-lowering medications: United States, 2005–2006 to 2013–2014 [Research Letter]. Circulation 2018;138:208–10. http://circ.ahajournals.org/content/138/2/208.

National Center for Health Statistics, National Health and Nutrition Examination Survey, 2015–2016. https://www.cdc.gov/nchs/nhanes.htm.

